# Bronchoscopy

**Published:** 1902-08-09

**Authors:** 


### The British Medical Association.

### BRONCHOSCOPY.

Among the most interesting and original papers read at this meeting was one contributed by Professor Gustav Killian, of the University of Freiburg, on the subject of endoscopy of the œsophagus and upper air-passages. He said that remarkable progress had been made during the last few years in the diagnosis and treatment of foreign bodies in these parts. An entirely new series of methods of examining these passages had been evoived to relieve us from the great difficulties which had been so frequently met with. He more especially alluded to certain methods of direct examination which are based on the recognition of the fact that we may penetrate in a straight line into the air-passages without damaging these organs. Direct œsophagoscopy may be employed in most patients by practised hands; as a rule local anæsthesia with cocaine is sufficient, but in excitable patients and with children general narcosis is necessary. By its means the gullet may be systematically searched from above downwards; we are able to recognise the exact place and form of the foreign body, its shape, its position, and its relation to the gullet wall, and we can see whether the latter is inflamed or has been injured by previous vain attempts at removal or pushing forward of the foreign body. We can then select an instrument suitable for extraction according to the size and consistence of the foreign body, and this can then be brought out either through the œsophagoscope, or, if too large, together with the latter instrument. If, however, the foreign body is large or has sharp edges, points, or hooks, as, for instance, in the case of dental plates, all attempts at extraction are dangerous, and in such cases œsophagotomy is indicated if the foreign body be situated no deeper than 24 to 26 cm. from the upper incisors. If deeper than this gastrostomy or posterior mediastinotomy is generally called for; although the author of the paper related a case in which he had succeeded in cutting up a large vulcanite plate by means of a galvano-cautery snare and removing it piecemeal. The same principle which lies at the foundation of œsophagoscopy has within recent times also been applied to the trachea and bronchi, it having been shown that it is possible to make an inspection right down into the lungs by the introduction of straight tubes into the trachea and bronchi, a fact most important for finding and removing foreign bodies impacted there. Ordinary laryngoscopy by means of the laryngeal mirror has had its triumphs, but all will acknowledge that just in those cases where foreign bodies most frequently occur, namely, in children, insurmountable difficulties often arise. In these cases with practice, especially after administration of an anæsthetic, the patient's head being in a dependent position, direct laryngoscopy (Kirstein's orthoscopy) can always be successfully employed. Of course if there be much dyspnœa a preliminary tracheotomy must be performed. Professor Killian has thus been able to remove from the larynx of a child a tightly fixed collar stud, which would otherwise have required a thyrotomy. Still more important is direct laryngoscopy in the case of impaction of foreign bodies in the trachea, as ordinary laryngoscopy more frequently fails in these cases. Pressure on the base of the tongue or the laryngeal surface of the epiglottis with one of the different spatulas designed by Kirstein may suffice to give, even in young children, a view into the trachea; but the author much prefers to employ direct upper tracheoscopy, using a straight tube of sufficient length and a width small enough to get through the rima glottidis. He is thus entirely independent of any disturbing reflex action of the pharynx or larynx. In the adult, local anæsthesia with a 25 per cent. alcoholic solution of cocaine is nearly always sufficient, but a preliminary injection of morphine in addition is a great help. In children it is best to give chloroform, the head hanging over the edge of the table. The instrument is introduced under the guidance of the eye, looking continuously through the tube and finding the way gradually through the pharynx and larynx into the trachea. Foreign bodies are thus easily discovered and extracted.

Aspirated foreign bodies are specially apt to become fixed in the bronchi, and if only just beyond the bifurcation direct tracheoscopy may be all that is necessary for their discovery and extraction. But if lower down, tubes of greater length may be employed and introduced into the cocainised bronchi. If tracheotomy has been necessary a short tube may be introduced through the tracheotomy wound into the cocainised trachea—lower direct tracheoscopy. "We may, without fear, press the bronchi, which are highly elastic tubes embedded in soft tissue, into the median line, and bring trachea, larger bronchus, and branch thus into one straight line. Thus out of the upper and lower tracheoscopy my methods of upper and lower bronchoscopy were evolved." Bronchoscopy allows the whole bronchial tree to be searched. Though the left principal bronchus branches off at a sharper lateral angle it is, through gentle pressure, brought almost as easily into view as the right. It must be remembered that if a tube is introduced into a principal bronchus which is entirely blocked by a foreign body, respiratory difficulties will at once occur, as air cannot pass in large enough quantity into the healthy lung by the side of the tube. Under such circumstances the tube should have a lateral opening at some distance from its lower end. Hitherto bronchoscopy has been employed in 20 cases, of which 11 were Professor Killian's own. Nearly one-half of all the cases were children. The foreign bodies were of the most varied character. In one-half of the cases they had been aspirated a few days before; in the other half they had been in the lung for months or years. In nine of the eleven in which upper bronchoscopy was employed, it led to certainty of diagnosis; while in all the nine cases of lower bronchoscopy it established the diagnosis. Extraction was attempted in 15 cases, and succeeded eight times with upper and five times with lower bronchoscopy. In two cases the attempt was unsuccessful, and both these patients died; but of those in whom extraction was successful only one patient died, and that nine months later from purulent pleurisy of the healthy side.

